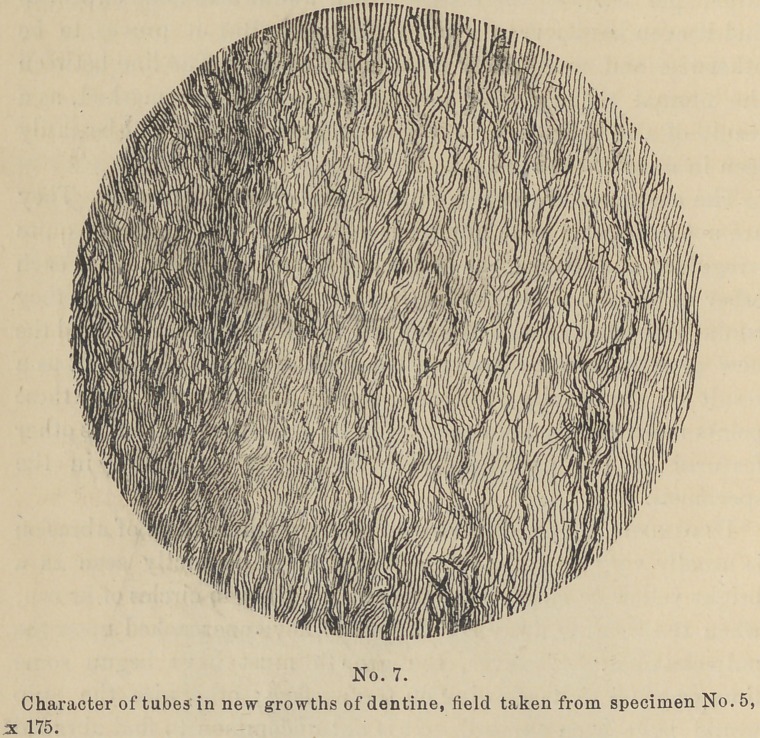# Protective Dentine, or Dentine of Repair

**Published:** 1888-01

**Authors:** M. H. Fletcher

**Affiliations:** Cincinnati, Ohio


### THE DENTAL REGISTER.

VOL. XLII.] JANUARY, 1888. [No. 1.

### COMMUNICATIONS.

### Protective Dentine, or Dentine of Repair.

BY M. H. FLETCHER, D.D.S., M.D., CINCINNATI, OHIO.

Read before the Section on Dental and Oral Surgery of the Ninth International Medical Congress, held at Washington, D. C., September 5th, 1887.

TERMINOLOGY. In the terminology of the pathological growth of the teeth known as Secondary Dentine, or Dentine of Repair, the two terms are used synonymously. But since the term, Secondary Dentine, may be, and is applied to a new growth of dentine whether in or out of the pulp-chamber and whether conservative or otherwise, this usage has become conventional. But this seems hardly the most specific name to give to the particular new growth which we wish to present. The term, Dentine of Repair, is applied to that growth of dentine which the pulp throws out, or builds up, in order to protect itself from an external enemy. But, since, in these cases, nothing is restored or repaired to a sound or good condition as is done in restoring the contour of a decayed tooth, the word "repair" does not seem the most appropriate. The writer proposes for this form of growth the name *Protective Dentine*, as being best suited to the conditions attending this formation. This term should be used in the place of "Dentine of Repair" and should be confined strictly to that growth which is intended to, and does, protect the pulp from exposure produced by external agents, and the term *Secondary Dentine*, should include all varieties of new growths of dentine whether in or out of the pulp-chamber. This being, as it were the specific name, and the other forms being varieties, the writer suggests, therefore, the following division in order to simplify the classification :

Secondary Dentine to include :

1. Protective Dentine.
2. General Deposit of Dentine within the Pulp-Chamber.
3. Dentinal Tumors either within or without the Pulp-Chamber.
4. Pulp Nodules of Dentine.

The unorganized calciferous deposits form another specific class.

This growth of protective dentine is clearly for protection and is to be found in most of the herbivorous animals as well as in man, when their teeth are sufficiently worn to need it.

This term may have been used formerly as above, but I have not yet met with it.

Definition. Protective Dentine then is that form of new growth of the hard tissues of the teeth characterized by its appearance within the pulp-chamber at a point where the pulp would have been exposed if the destructive process which excited the growth had been continued. (See cut No. 1.)

No. 1.

Vertical section of deciduous canine. D, dentine, E, enamel, P, pulp chamber C, cavity of decay, Pd, protective dentine. x 15.

Another characteristic which is common to this, as well as other new growths of dentine is, its comparatively few and somewhat irregular dentinal tubes and a larger per cent. of globular masses or calciferous material than is normal. We also find between the irritated surface of the tooth and the surface of the pulp, a zone in which the dentinal tubes are greatly reduced in size by a deposit of lime salts in their walls, in consequence of which the matrix of the dentine is much greater in proportion than is normal. This gives it a dense, horny appearance and makes it quite translucent. The portion of dentine thus affected is called by Magitot the "Zone of Resistance. (See cut No. 2.)

No. 2.

Transverse section of lower first molar, just above neck of tooth, having had large amalgam filling on approximal and crown surfaces. D. dentine, Zr. Zone of Resistance, St, small tumor, T, predunculated tumor almost filling pulp chamber P, pulp chamber, B, where the tumor was broken from its attachment or seat of growth. x 15.

CLASSIFICATION. New growths of the pulp and pulp-chamber are primarily of two classes: those which fasten themselves to the inner wall of the chamber, called adherent, and those which are found in the pulp itself, called unattached. Dr. Black classifies and defines them as follows:

1. Secondary Dentine. A new growth of dentine more or less regular in formation.
2. Dentinal Tumor within the pulp chamber.
3. Nodular calcifications among, but not of, the tissues of the pulp.
4. Interstitial calcification of the tissues of the pulp.
5. Cylindrical calcifications of the pulp.
6. Osteo-Dentine.

This is the most comprehensive classification the writer has met with, and yet it does not give any definite character to that particular growth which is so prevalent as to be almost physiological and without which many teeth would become useless.

Etiology. In the etiology the chief cause seems to be the disturbance of the pulp through the dentinal fibrils, the irritant being at the peripheral ends. This irritation may be caused by any thing which persistently disturbs the ends of the fibrils, such as caries, fillings, abrasions, clasps, etc. Garretson says, "Teeth subject to sources of local irritation are frequently—indeed it is to be said—are commonly found responsive in the way of self-attempting deposits." He also says, "No substance introduced into a tooth seems to exert greater influence in the excitation of that action which produces secondary dentine than does oxychloride of zinc." And it is a practice highly thought of, and followed by many practitioners, to stimulate in every known way, a deposit of new dentine when a pulp is exposed or approximates an exposure. Some English amalgams are supposed to possess this stimulating property to a marked degree, and are used by many with that object in view. But from observation of final results this seems to be a pernicious practice. We have some specimens to present which illustrate this point. They show that not only Protective Dentine has been induced, but a general deposit of Secondary Dentine sufficient to reduce the chamber very greatly in size with pulp nodules in addition, and that to the degree of the loss of the teeth. (See cuts No. 2 and 3.)

No. 3.

Vertical section of lower first molar, D. dentine, P. pulp chamber, F. amalgam filling In Situ, Pd, protective dentine caused by irritation of filling, E. enamel. x 35.

Enough of such specimens can be presented to prove almost conclusively that any thing which constantly irritates the distal ends of the dentinal fibrils—providing it is not too rapid and destructive in its progress—is likely to stimulate not only one but all of those new growths and calcareous deposits mentioned. My own observations show that large amalgam fillings exert a greater influence in stimulating new growths than gold. Nevertheless, this stimulation seeme to come largely from thermal changes conveyed to the pulp through the metal.

The influence, however, does not always stop with the particular tooth irritated, but may extend to any or all of the others.

Dr. Black speaks of this effect and likens it to a certain disease of the eye, presumably Sympathetic Ophthalmia, in which case the offending organ is frequently enucleated in order to save the sight of the good one. If it were possible to arrest this new growth just when one would like, its intentional stimulation would be a most excellent procedure. But investigation seems to prove that the final result is generally evil rather than good. However, it should be remembered that a growth of Protective Dentine in many cases (abrasion especially) extends the period of usefulness of the teeth to a marked degree. While this conservative process is most desirable up to a certain point in the human teeth, there is a point at which it should be arrested in order to still prolong their usefulness.

No. 4.

Vertical section of a deciduous canine. Pd, 1, shows protective dentine from abrasion of crown marked A. C, cavities of decay, Pd, protective dentine caused by decay on approximal surface, P, pulp chamber, D, dentine. x 15.

PATHOLOGY. In the pathology of this growth, we find on the surface of the tooth some lesion. As above mentioned this may be any thing which constantly irritates the [distal ends of the dentinal fibrils. At an early stage of the lesion the Zone of Resistance can usually be found forming in that portion of the dentine lying between the lesion and the pulp adjacent. (See cut No. 2). Within the chamber we find in apposition to that portion of the pulp which comes in contact with those dentinal fibrils whose ends are exposed to the irritant, a deposit of Protective dentine. (See cuts Nos. 1, 2, 3, and 4).

If the exciting cause is abrasion, that cornua of the pulp-chamber nearest exposed, is the first place of deposit. (See cut No. 4 Pd 1). If the irritation is caused by filling, decay, or erosion, the point at which the pulp is nearest the irritant is the first to show the new growth.

No. 5.

Vertical section of same tooth as No. 6, E. Enamel, D. Dentine, Sd. Secondary Dentine, P. Remains of Pulp chamber even with neck of the tooth. At which stage the pulp became exhausted and died. x 15.

In the beginning the growth is confined to those fibrils thus affected, but progresses in proportion to the lesion, up to a certain degree. If the irritation continues, the deposit becomes more and more general until the whole membrana eboris is excited to an abnormal amount of work, and the result is, a narrowing down of the size of the pulp-chamber from a general deposit of Secondary Dentine within the walls (see cuts Nos. 5 and 6) or as nodules or as deposits of calcareous material or, it may be, all of these forms. In numbers of these cases the pulp dies, but whether as a consequence of simple compression or, as seems to me more probable, by reason of the occlusion of a large portion of the blood-vessels owing to atheromatous and calcareous deposits in their walls, is not yet determined, but probably will be on further investigation. We have some examples of these narrowed pulp-chambers to show. (See cut No. 5).

No. 6.

Transverse section of lower central incisor, one half of crown worn away by abrasion. D, dentine, E, enamel, Sd, secondary dentine entirely filling the pulp chamber with the exception of the small space at P, x 15.

This substance which is deposited for protection is built much more rapidly than the normal tissue, and is inferior in quality. It has a much smaller per cent. of tubes and a proportionally larger per cent. of the matrix which would make one expect to find it even harder than normal dentine. But it proves to be otherwise and wears away much more easily. The line between the normal tissue and the new growth is distinctly marked, as a result of the difference in their structure, which will be easily seen in most of the sections.

The character of the tubes in the new growth is distinct. They are not so regular or so numerous and, in fact, at times are quite irregular, and their courses are not so apt to be parallel to each other as in normal Dentine. (See cut No. 7). Many times they run at right angles with the normal tubes (See cut No. 5) and the new growth seen by transmitted light is quite translucent as a result of the paucity of tubes. But as a section will show these points more plainly than they could be told, they as well as other features of the pathology will be left to be shown in the specimens.

Diagnosis. The diagnosis of this growth in cases of abrasion is usually very easy, for the new growth is distinctly seen as a bright yellow or clear spot, (See cut No. 6) or in circles of brown, when the wearing away is sufficient to have encroached upon the pulp-chamber. However, the growth must have begun some time previous to its showing on the surface; otherwise the pulp would have been exposed. It is not uncommon to find abraded teeth quite sensitive to the touch of steel or other hard substances. This sensitiveness probably arises from the dentinal tubes not having been sufficiently filled by the process which produces the Zone of Resistance, or it may come from the rapid work of the destructive agent, it being such that the pulp can not build the protection fast enough to shield itself entirely from the disturbing influence. Hence, when we find a tooth worn down into close proximity to the pulp, we may be reasonably sure that there is a deposit of Protective Dentine going on in that tooth. The amount of wear may be determined by comparing the present length of the crown with what it originally was. Erosion may be judged in much the same manner as abrasion. If shallow, there is probably very little or no growth at all; if deep, the deposit is apt to be in proportion to the extent of the erosion.

No. 7.

Character of tubes in new growths of dentine, field taken from specimen No. 5, x 175.

To diagnose in caries is much more difficult, since there is usually no pain attendant with these growths so long as the pulp retains its full vitality; nor can the deposit be easily seen like it can in abrasion. Besides, the decay is in many cases so rapid that the pulp has not sufficient time to make a deposit of any considerable amount before its surface is reached. Much can be determined, however, from the general appearance of the tooth in connection with other points. For instance, if the decay is dark and hard and almost stationary instead of rapid in its progress, and the patient is forty or over, new growths are most likely to be present, at least the protective growth, since, at the age of forty, they are most commonly found. Dr. Black says that he "Occasionally finds them in those not yet past their teens." And Salter says "Dentine of Repair sometimes occurs in temporary teeth."

The writer has found it in a majority of deciduous teeth, when they have been retained up to, or past their normal time and are much decayed or abraded. (See cuts Nos. 1 and 4). But I have found no nodules or other new growths in connection with the Protective Dentine. It seems evident they would follow, however, if the life of the tooth was not destroyed by natural processes. An approach to the spontaneous arrest of decay is most frequently found in what are recognized as a good quality of teeth. Abrasion is also found in teeth of this kind, and it is in just such teeth that we may expect to find, not only Protective Dentine, but many of the other forms of new growths.

Prognosis. In the prognosis of these cases we come to the practical part of our subject. For if we can tell what will be the result of these growths, we are the better able to decide what to do in order to prevent evil results. From the foregoing (as has been stated) it is evident that this building of Protective Dentine is a provision of nature for the express purpose of protecting the pulp from a recognized enemy; hence the names which have been given it: "Secondary Dentine," "Dentine of Repair," and "Protective Dentine." In cases where musket balls have been shot into the pulp-chamber of an elephant's tusk, they have become perfectly encapsuled with ivory—a specimen of which will be shown. From this, and the other specimens of protective growths, we may get some idea of what a tooth pulp will attempt to do, if called upon for such work. The elephant's tusk is a tooth of persistant growth, and the formation of Dentine goes on about the bullet until it is completely encased, and is carried out of the skull with the tusk as it proceeds in its growth. The formation of Dentine is carried on by the same process in man as in other mammals. But the human tooth is of limited growth, and if the process of forming more Dentine within its chamber is continued abnormally, it can only be done at the expense of the pulp, and this to its own peril. For it must be understood that this process of repair once established in a human tooth, appears in many cases to be attended by, or to result in, the death of the pulp. Whether this is a result of a purely local condition of the pulp-chamber, or of a disease of the blood-vessels, remains to be determined, as stated in a preceding portion of this paper. In many cases the pulp begins to be exhausted and show symptoms of death while there is yet considerable pulp-chamber left (see cut No. 5), and it seems that there is, in all these cases, more or less of the chamber remaining unoccupied by the new growth, whether adherent or unattached. It never has been my experience to see a "complete dentification of the cavity of the pulp" as is mentioned by Magitot, Owens, and others. The writer is convinced that such a condition can not exist, since the pulp must have room for its necessarily constituent parts in order to form any of its products; The utmost limit of space to which a pulp can be contracted and yet live, is large enough, when the pulp dies, to form a reservoir for putrid matter sufficient to cause an alveolar abscess which is usual, providing the apical foramen of the tooth does not become occluded by the debris of the dead pulp. The amount of space thus remaining varies in different teeth, and different persons. With this morbid condition of affairs at hand we may have all the symptoms usually present with a dying or dead pulp. The writer is convinced that when pain accompanies new growths of the pulp-chamber, it is not caused by their presence there, or by their pressure on the pulp from their size or quantity, but from the inflammation excited by the presence of a necrosed pulp. Hence it seems safe to conclude that if means are not taken to stop the progress of these growths in their incipiency, evil will result in many cases sooner or later.

Treatment. It is the mission of the profession, not only to repair and replace broken-down tissues, but to institute prophylactic treatment when it is indicated. The treatment then in these cases to prevent evil results would be the removal of the exciting cause. If the cause be caries, plug the cavity in such a way that the filling is non-irritating. If it is abrasion or erosion, stop its progress with a gold capping. If a pulp has been exposed, it would seem a bad practice to lift more of the dentine and make the exposure larger in order that a substance may be applied, supposed to excite a new growth of dentine. If the pulp lived under these circumstances to deposit new material it would do so only to protect itself from the work thus done. The rational treatment then in such a case would be the sterilizing of the softened dentine with a suitable germicide and the careful adjustment of a non-iritating cap to both pulp and dentine before filling. By adopting the above methods we would hope to avert destructive processes and not to excite dangerous new growths.

## Figures and Tables

**No. 1. f1:**
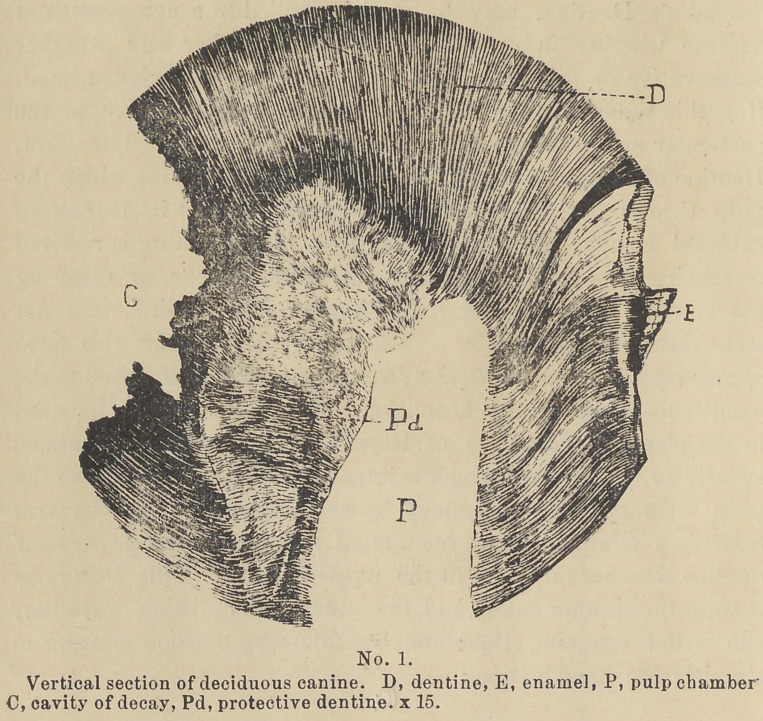


**No. 2. f2:**
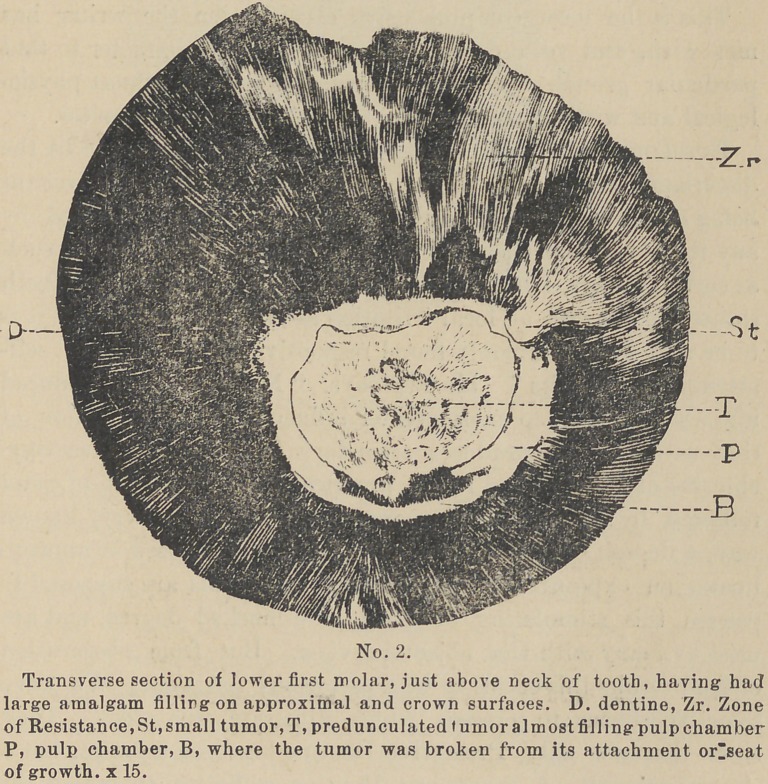


**No. 3. f3:**
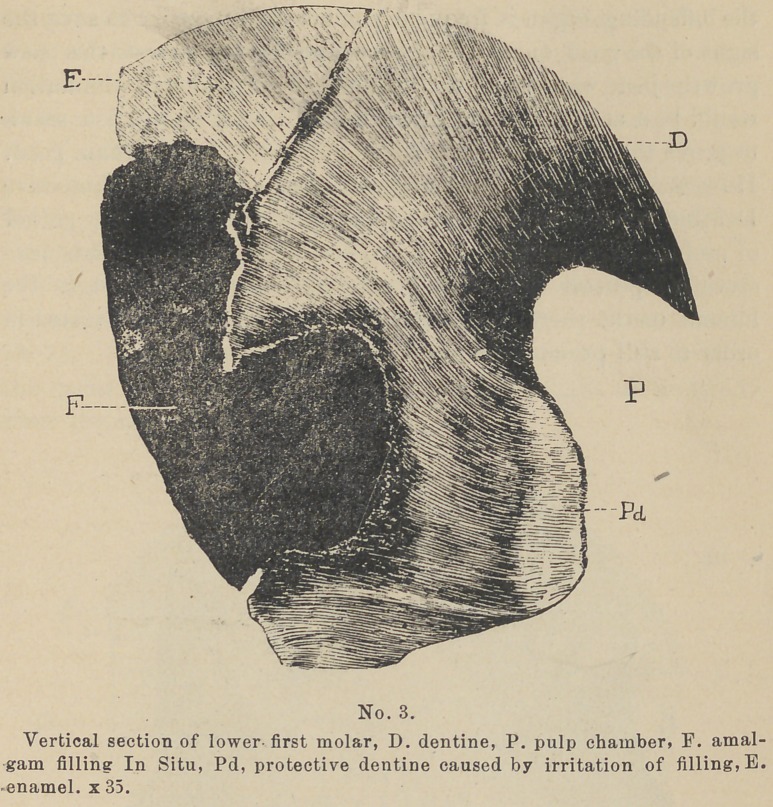


**No. 4. f4:**
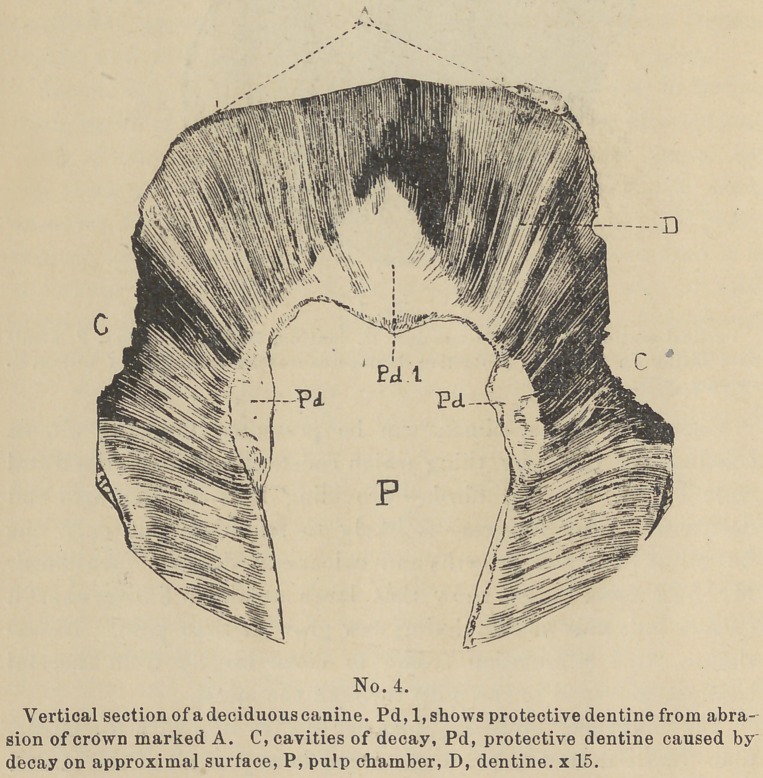


**No. 5. f5:**
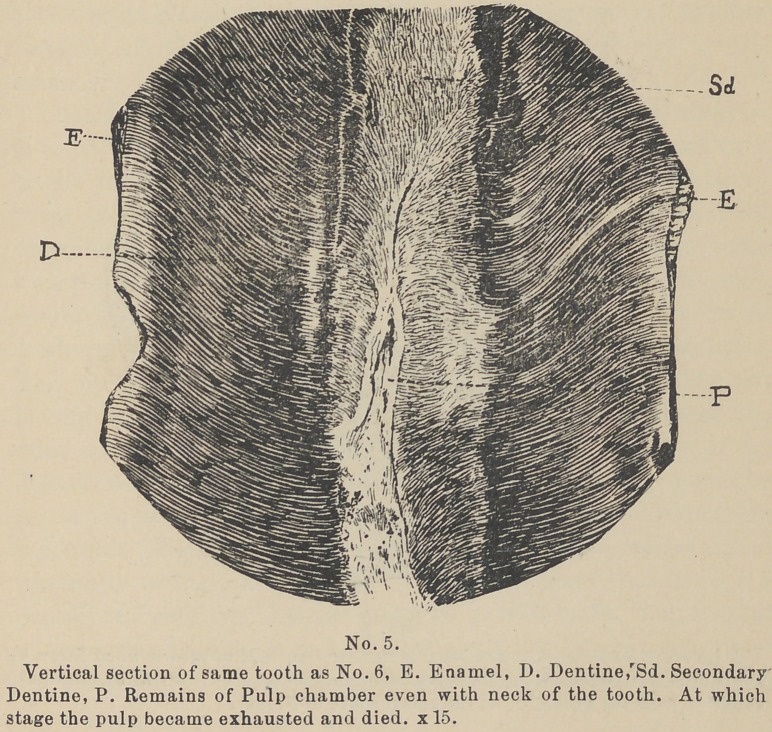


**No. 6. f6:**
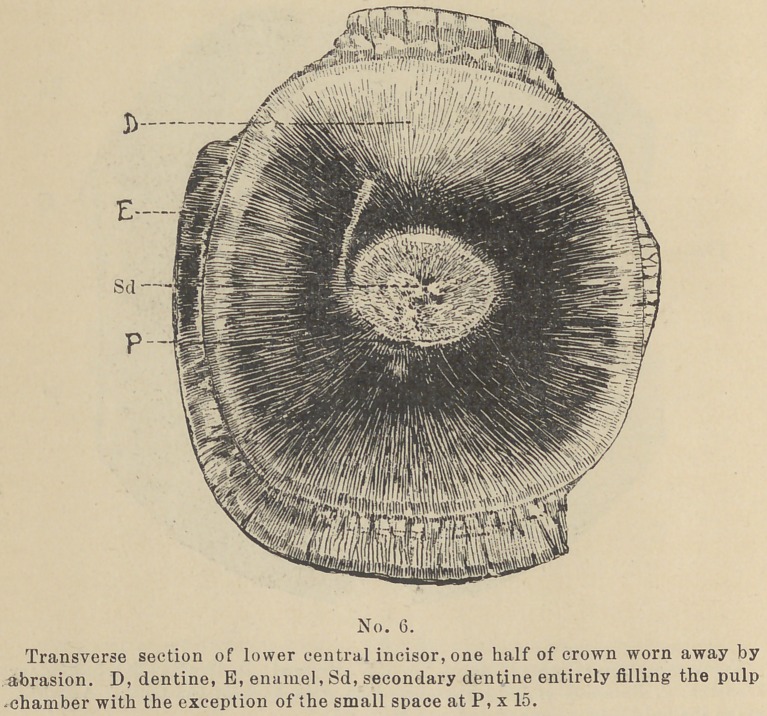


**No. 7. f7:**